# Ocular implants-methods of ocular reconstruction following radical surgical interventions

**Published:** 2018

**Authors:** Corina Teodora Catalu, Sânziana Luminiţa Istrate, Liliana Mary Voinea, Costin Mitulescu, Viorela Popescu, Ciuluvică Radu

**Affiliations:** * Ophthalmology Department, University Emergency Hospital, Bucharest, Romania; ** Anatomy Department, “Carol Davila” University of Medicine and Pharmacy, Bucharest, Romania

**Keywords:** ocular implant, evisceration, enucleation, exenteration, orbital reconstruction, hydroxyapatite, polyethylene, postoperative complications

## Abstract

The main motivation of an ocular-orbital reconstruction after a radical surgical intervention (evisceration, enucleation) is represented by the psychological and socio-economic impact of such interventions on life conditions of patients. The current methods for ocular prosthesis are based on a new concept, which is nanotechnology, and its main objectives represent the reconstruction of the remaining orbital volume, reduction of postoperative complications and maintaining a satisfactory esthetical aspect.

This review will discuss the numerous types of ocular implants that have been used throughout history as well as the most recent methods used by ophthalmic surgeons, also taking into consideration the advantages and disadvantages from a cosmetic, functional and short and long term postoperative complications point of view.

## Introduction

The psychological support of the patient pre and post-operatively is important after a radical intervention. It is fundamental that in such cases, the patients’ re-adaptation to social life and the esthetic aspect is taken into consideration.

There has been an increasing desire to create the ideal ocular implant as well as to improve surgical techniques, in favor of reducing the frequency of postoperative complications. Different materials have been used to improve the biocompatibility and aesthetic appearance of an ocular implant (fat, bone fragments, cartilage, silver, titanium, glass, porous materials, silicone, etc.) for more than a century [**1**-**3**].

When discussing the biocompatibility of a certain nanostructured material, we have to consider 3 aspects: bio-adaptability, bio-tolerability and bio-functionality [**4**]. Progress in this field led to improved designs of ocular implants. 

An ideal ocular implant should be non-allergenic, nontoxic, not provoking host tissue immune response, mechanically stable with satisfying motility and a suitable quality to price ratio [**5**].

For improved motility of ocular prosthesis, most surgeons attach the extrinsic muscles to the ocular implant also reducing the risk of exposure or extrusion. This is why types of implants integrated and non-integrated are being discussed [**6**]. 

**General considerations of orbital anatomy**

The orbit is a pyramid-shaped cavity, with the anterior base and the posteromedial apex, and four walls: lateral, medial, floor and the orbital roof. There are communications to the orbit with neighboring regions through orifices located on the orbital walls. Due to low resistance areas, there is a high frequency of fractures located in the zygomaticomaxillary and zygomaticofrontal sutures. Fractures of the orbital plane and the medial wall are common in diffuse traumas [**7**-**9**].

Anatomically speaking, there is a space between the orbit and the periosteum that allows the ablation of the orbital content. Orbital content consists of the eyeball with its annexes (Tenon capsule, conjunctiva, lacrimal apparatus, eyelids, oculomotor muscles), optic nerve, ophthalmic artery, ophthalmic vein, peripheral nerves, periorbital fat [**10**].

The adult orbital volume is about 30 cm³ and has a depth of 45-55 mm. Constitutionally, the male orbit is larger than in women. Also, the Broca index, which represents the ratio between the orbital cavity height and its length, is very variable depending on race [**11**-**13**].

**Radical interventions: surgery**

**Fig. 1:**
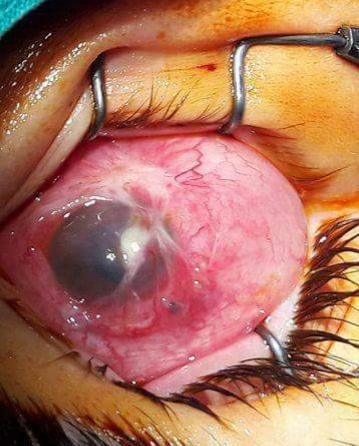
Post corneal fungal ulcer with a conjunctival flap

**Fig. 2:**
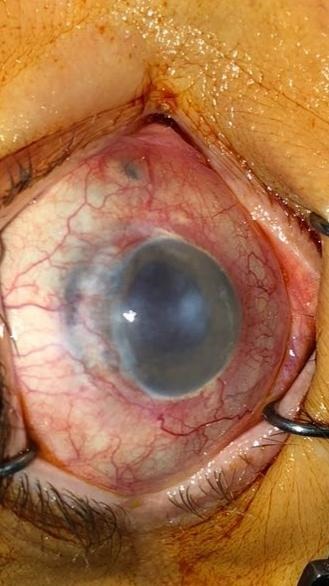
Neovascular glaucoma

Depending on the ocular pathology (**Fig. 1**,**2**), the removal of the eyeball can be accomplished by three surgical techniques, namely: evisceration, enucleation or exenteration. There are pros and cons in applying these surgical methods, but the main purpose remains the preserving of the patient’s life and the increase of the quality of life through various methods of ocular prosthesis.

Regardless of the surgical technique, there is always a preoperative assessment, taking into account the history of ocular pathology (congenital or acquired ocular affection), objective and paraclinical examination, patient requirements (discomfort, aesthetic aspect), associated general disorders, explanation of pathology and surgical technique (pre- and post-operative psychological counseling), and, last but not least, written consent of the patient [**14**,**15**].

Evisceration and enucleation can be performed under local or general anesthesia, the latter having the advantage of not modifying the anatomy of the orbital vicinity [**16**].

**Fig. 3:**
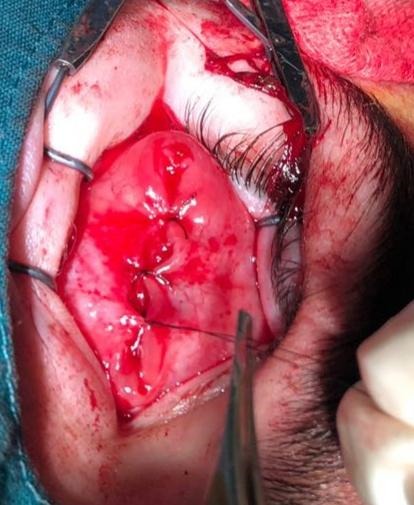
Evisceration, one of radical surgical interventions

Moreover, evisceration is a procedure in which the globular content is removed, keeping the sclera, Tenon capsule, conjunctiva, extrinsic muscles, and the optic nerve (**Fig. 3**) [**17**]. Enucleation is another surgical option represented by removing the ocular globe and keeping only the bulbar conjunctiva and extrinsic ocular muscles [**16**,**18**].

Evisceration has been considered superior to enucleation for a long time, due to the esthetical aspect and motility, but current surgical methods suggest the attachment of the extrinsic muscles at the implant after an enucleation [**18**,**19**].

Exenteration is a more complex procedure involving collaboration in a multidisciplinary team (ophthalmologist, plastic surgeon, neurosurgeon, oncologist, ENT), and involves removing the eyeball with its attachments and the orbital content of the periosteum that associates the eyelid removal or not [**20**].

In cases of intraocular tumors, i.e. melanoma or retinoblastoma, evisceration is contraindicated due to high dissemination risk [**16**,**21**,**22**].

**Table 1:** Radical intervention indications [**20**-**22**]

EVICERATION	neovascular glaucoma, infectious endophthalmitis, disorganized globe, eye injury without uveal involvement, corneal perforation ulcers, etc.
ENUCLEATION	intraocular tumors without local extrascleral invasion (melanomas, retinoblastomas), painful eyes with important damage to VA, the need of histopathological examination, important ocular traumas with significant uveal involvement, risk of sympathetic ophthalmia, etc.
EXENTERATION	malignant ocular tumors (palpebral spine cell carcinoma, conjunctival or lacrimal sac, basal cell carcinoma, adenocarcinomas, melanomas, rhabdomyosarcoma, etc.); aggressive benign tumors (meningiomas, gliomas, etc.); vascular facial malformations, massive orbital varicose veins, orbital pseudotumor, aggressive orbital mycosis, trauma orbital causing orbital cellulite that does not respond to treatment, etc.

**Fig. 4:**
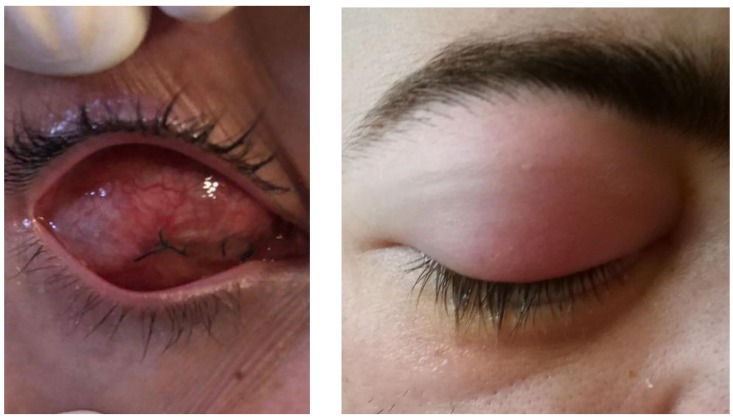
One day after evisceration (palpebral edema)

Complications occurring either intraoperatively or postoperatively (**Fig. 4**, **Table 2**) should be considered. In case of evisceration, there is a risk of local intraoperative hemorrhage, especially when a coagulation disorder is associated, as well as the risk associated with general or local anesthesia [**20**].

In terms of enucleation, intraoperative incidents are uncommon, but the following are possible: puncture of sclera in patients with malignant intraocular tumors, loss of extrinsic eye muscles in orbital fat, etc.

Sino-orbital fistula is a complication quite commonly encountered intraoperatively in the exenteration approach, and dural rupture is a vital risk complication involving extrusion of the cerebrospinal fluid [**23**].

**Table 2:** Different postoperatory complications [**15**,**20**,**22**,**23**]

Evisceration	Enucleation	Exenteration
- extrusion of the ocular implant	- extrusion of the ocular implant	- postoperative infections (especially in the neglected sino-orbital fistula)
- postoperative infections	- postoperative infections	- tumor recurrence (most important)
- increased eye pressure	- palpebral edema (**Fig. 4**)	- delayed healing (frequent in patients post-radiotherapy or those with diabetes)
- local pain (due to corneal preservation)		- graft failure
		- cutaneous and mucous hypoesthesia (often by infraorbital nerve injury, nerve supraorbital and anterior and posterior ethmoidal nerve.

The procedure ends by placing an ocular implant in the ocular cavity. In the case of ocular integrated (HA) implant, prosthesis is placed in about 6 months post-op, prior to which, a CT scan is performed by visualizing the fibrovascular invasion of the mentioned implant [**24**,**45**].

**Types of ocular implants**

The search for an ideal ocular implant also led to a progress regarding the surgical procedure as well as reducing post-operative complications after an evisceration or enucleation. For instance, Sami et al. mentioned three categories based on the nature of each type of implant: buried, exposed-integrated, and buried-integrated implants [**6**]. Integrated ocular implants offer the appropriate dimensions unlike the ocular prosthesis that has a volume of 4,2 ml less than the volume needed for ocular reconstruction [**26**,**27**]. 

Regarding the silicone sphere implant, it has been used for over 50 years [**30**]. Many surgeons argue that it would be a less favorable choice if it were implanted without attachment of extrinsic muscles, due to extrusion risk and reduction in motility [**28**,**29**].

Looking at the chemical, physical, and structural characteristics of orbital implants, comparative studies on such topics are quite rare in the literature. It has been recognized that adequate fibro-vascularization is vital for a porous implant to achieve long-term success: chemical composition, microstructure, and mechanical features are all factors that play a role, but there is a wide variation in these characteristics among the available materials [**31**].

***Hydroxyapatite***

Porous orbital implants spread worldwide after the introduction of modern HA orbital implants, which are not based on treated bone derived from animal sources. HA formally belongs to the class of calcium orthophosphates and, especially in the form of coralline or synthetic HA, has been widely used for more than 50 years in orthopaedics and dentistry for bone repair, thanks to its chemical and compositional similarity to the biological apatite of hard tissues [**32**–**34**]. 

In the mid-1980s, Perry [**35**] experimentally introduced the coralline porous HA sphere and it has been commonly adopted in clinical practice since the early 1990s, eventually becoming the most frequently used implant after primary enucleation [**36**]. Porous HA implants have been widely studied, and many retrospective reviews on patients’ outcomes are available in the literature [**28**]. The interconnected porous structure of the HA implant allows host fibrovascular in-growth, which potentially reduces the risk of migration, extrusion and infection [**37**,**38**].

In a prospective study of 60 patients with different ocular pathologies, it was found that 14% of the patients with primary ocular implant showed ocular implant extrusion and only 1% of the patients with secondary ocular implants. Of the 60 patients included in the study, 27 received primary ocular implant after enucleation and one after evisceration, and 32 ophthalmic patients received secondary implant [**39**].

HA implant includes the intrinsic cost of the implant, which is often the most significant cost – the need for a wrapping material, the assessment of implant vascularization with a confirmatory MRI study and, optionally, a secondary drilling procedure for peg placement with the consequent modification of the ocular prosthesis [**40**].

Lower-cost versions of these materials have been developed and are currently in use around the world. Therefore, it is generally recommended that HA implants are placed within a wrapping material before being introduced into the orbit [**24**,**25**,**41**]. It was shown that the majority of the exposed HA implants can be successfully treated by using patch grafts of different origin (e.g. scleral graft, dermis graft, oral mucosa graft) without the need for implant removal [**42**-**45**].

Youn-Shen Bee et al. have shown in a study that the increased number of preoperative leukocytes may be associated with the increased risk of extrusion of the ocular implant (approximately 13%). This study was conducted on 85 patients with ocular infections in whom radical surgery was performed (evisceration or enucleation) [**46**]. 

In the case of orbital implant infections, administration of systemic antibiotics and topical eye drops can solve the problem, but if no improvement in the symptoms is noticed, implant removal should be considered [**47**].

Other reported complications include conjunctival thinning (followed or not by exposure), socket discharge, pyogenic granuloma formation, mid-term to chronic infection of the implant, and persistent pain or discomfort [**45**,**48**,**49**].

In summary, porous HA implants remain the most commonly used in anophthalmic surgery, together with their advantages and suitability [**50**]. However, in the search for an “ideal” porous orbital implant with a reduced complication profile and diminished surgical and postoperative costs, alternative materials have been also explored over the last two decades.

***Poly (methyl methacrylate)***

PMMA is known in ophthalmology as well as rigid and semi-rigid contact lenses [**51**], due to its excellent biocompatibility with ocular tissues and transparency to visible light.

In 1976, Frueh and Felker first described the use of the so-called “baseball implant”. Although originally described as a secondary implant, its design might allow primary implantation as well [**52**].

In 1985, Tyers and Collin implanted 35 secondary and six primary baseball implants and monitored the patients over a 24 months follow-up period [**53**]. Complications occurred in 59% of the cases, but most of them were resolved by pharmaceutical treatment. The authors concluded that the baseball implant showed good potential and might be recommended both as the secondary implant and as the first approach to a volume deficit in the anophthalmic socket. On the other hand, they acknowledged that the reported series of primary baseball implants were too small to allow them to draw definite conclusions [**52**,**53**].

In 1994, Leatherbarrow et al. [**54**] reviewed 44 patients receiving the baseball implant and reported six cases of severe complications (unacceptable pain, implant migration and implant exposure). In the late 1990s, Christmas et al. [**55**] implanted the baseball implant in six patients (primary enucleation) and after 14 days appeared as exposure of ocular implants.

In summary, PMMA is an excellent biomaterial for ophthalmic applications. In an interesting study, Groth et al. [**56**] treated nine severely injured patients implanting CT-based biomodelled, prefabricated, heat cured PMMA implants that were well tolerated postoperatively.

***Polyethylene***

The advantages of porous polyethylene are mainly the low cost in comparison to HA and the possibility of suturing extrinsic muscles directly from the implant. It has been used in patients with facial deformities, orbital fractures, but also from the aesthetic point of view.

In an animal model study, Goldbag et al. [**57**] showed that the HA implant induces a high risk of inflammation and local fibrosis versus porous polyethylene.

In a study of two patient groups, Sadiq et al. [**58**] found that the rate of postoperative complications after implantation of an HA or PE was similar, but the PE implant showed better motility.

By looking at the future of PE orbital implants, Kozakiewicz et al. [**59**] implanted ultrahigh-molecular-weight PE implants in three patients with orbital reconstructions. Based on the CT scanning, Kozakiewicz et al. prepared a virtual model of both orbits (injured and uninjured). The two resulting surfaces were then overlapped and the outer surface was used to design the external surface of the implant. This new procedure could also be applied in the future for the design and manufacture of orbital implants that closely mimic the original shape and size of the anophthalmic socket [**59**,**60**].

***Quasi-integrated implants***

In terms of fibrovascular in-growth and motility, Girard and co-workers [**61**,**62**] merged the advantages of porous and quasi-integrated implants for the first time. 

Guthoff et al. [**63**] developed a composited implant composed of two parts (anterior part made of synthetic porous HA and posterior part made of silicone rubber). The eye muscles were sutured crosswise in front of the implant to guarantee a better motility and stability [**64**,**65**]. This type of ocular implant is considered a good option, especially in Europe.

A preliminary study of 24 patients showed no cases of “quasi” implant extrusion and one of ten cases of enucleated pediatric patients had complications, but not significant [**66**]. 

***Aluminium Oxide***

Aluminium oxide (Al2O3) has been used for decades in orthopaedics thanks to mechanical properties, biocompatibility, and bio inertness [**67**]. Since the late 1990s, alumina has also been proposed, in a porous form, and was approved by the US Food and Drug Administration in 2000 and has been marketed under the commercial name of “Bioceramic implant”. 

Morel et al. [**68**] evaluated the clinical tolerance of porous alumina implants implanted in 16 eviscerated rabbits and only one infection was observed. Fibrovascular in-growth occurred as soon as 15 days postoperatively and was full at 1 month. Two years later, Jordan et al. [**69**], confirmed a comparison between the performances of alumina and HA implants. 

After reviewing a clinical case series of 419 patients, who received a Bioceramic orbital implant, Jordan and co-workers [**70**] showed that alumina implant infections are generally rare, with the majority of the exposures occurring after a 3 months follow-up period [**13**].

In a recent study, Ramey et al. [**71**] compared the complication rates of HA, porous PE and polyglactin-wrapped alumina implants and, interestingly, found that porous PE and alumina devices were associated with higher exposure rates and higher overall complication rates compared to HA implants; these results seem to contradict those reported by the majority of authors [**22**,**71**,**72**].

Zigiotti et al. [**73**] described a surgical procedure to reduce postoperative complication following alumina implant insertion in enucleated patients. The Bioceramic implant was covered with the patient’s sclera at the front. Over 16 months follow-up period no cases of implant extrusion with a good cosmetic result were present after final prosthetic fitting.

## Conclusions

Until present, a diversity of ocular implants has been used, some having the advantage of a good quality to price ratio.

Some studies observed that porous integrated implants (hydroxyapatite) might have complications like extrusion, dehiscence, or infections.

The use of integrated HA implant is preferred for enucleated patients, which allows a better integration of the ocular prosthesis resulting in good motility.

Developing the concept of nanostructured biomaterial holds a great interest for its ability to satisfy the need of an “ideal” ocular implant with higher healing and proliferation rate, low post-op complications (short/ long term), and an affordable cost.
